# Moderator's view: after SGLT2i and MRA antagonists, where do we go?

**DOI:** 10.1093/ckj/sfae013

**Published:** 2024-01-31

**Authors:** Carmine Zoccali, Jürgen Floege

**Affiliations:** Renal Research Institute NY, USA; BIOGEM, Ariano Irpino, Italy; IPNET, Reggio Calabria, Italy; Division of Nephrology and Rheumatology, RWTH Aachen University Hospital, Aachen, Germany

In this *CKJ* debate, Pantelis Sarafidis, Matias Trillini and Piero Ruggenenti provide complementary views of the current advancements and challenges in managing chronic kidney disease (CKD).

Pantelis Sarafidis underscores the increasing prevalence of CKD globally, highlighting the major public health concerns associated with it, such as kidney failure and heightened cardiovascular mortality. He primarily focuses on the traditional treatment approaches, including lifestyle modifications and pharmacotherapy with angiotensin-converting enzyme inhibitors or angiotensin-receptor blockers, which have been somewhat effective in slowing the progression of CKD, but have not completely addressed the issue and still are not in universal use in CKD patients, particularly those with increased albuminuria. He highlights that recent developments in CKD management have shown promising results by adding sodium–glucose cotransporter-2 inhibitors (SGLT2i) and non-steroidal mineralocorticoid receptor antagonists (MRAs) like finerenone. Unquestionably, these therapies have significantly improved kidney and cardiovascular outcomes in CKD patients with or without type 2 diabetes. SGLT2i have effectively reduced the annual loss of estimated glomerular filtration rate (eGFR) in patients with albuminuric CKD to lower rates than those associated with ageing. This suggests that with proper implementation of current treatments, most CKD patients can potentially reach a therapeutic ceiling.

Matias Trillini and Piero Ruggenenti delve into an articulated series of frontline therapeutic strategies and trials for nephroprotection. They discuss the SONAR trial's enrichment strategy using atrasentan, which successfully reduced the progression to serious kidney endpoints without significantly increasing heart failure hospitalizations (even though there was a trend towards more heart failure events with atrasentan). The trial's approach of pre-selecting patients responsive to treatment could inform future studies and optimize patient outcomes. Sparsentan, another promising therapy, is highlighted for its dual action as an angiotensin and endothelin antagonist, showing favorable results in reducing proteinuria and slowing eGFR decline in patients with immunoglobulin A nephropathy. However, sparsentan failed to affect the course of patients with focal segmental glomerulosclerosis. Furthermore, they emphasize the potential of personalized medicine in treating glomerular diseases like membranoproliferative glomerulonephritis through cluster analyses. By categorizing patients based on clinical features and complement system abnormalities, tailored interventions can be designed to target disease-specific mechanisms. This method has revealed distinct patient clusters with varying risks and potential treatment pathways. Matias Trillini and Piero Ruggenenti remark that cell therapy with mesenchymal stromal cells (MSCs) is a revolutionary approach to CKD treatment, offering a multifaceted mechanism to combat kidney injury. Clinical trials like NEPHSTROM have shown that MSC therapy can slow eGFR decline in CKD patients, indicating its safety and efficacy. Lastly, they touch on autosomal dominant polycystic kidney disease (ADPKD), a common genetic renal disease excluded from many trials. They mention successful trials using octreotide long-acting repeatable and tolvaptan for ADPKD management and hint at ongoing studies exploring various other treatments.

In summary, while significant strides have been made in CKD treatment with SGLT2i and MRAs, there remains a substantial portion of the CKD population at high risk for renal and cardiovascular events. Apart from the drugs discussed by Trillini and Ruggenenti, another innovative approach involves the use of glucagon-like peptide-1 receptor agonists. Originally developed for the treatment of type 2 diabetes, these drugs induce marked reductions in body weight in obese patients and have shown nephroprotective effects [1]. Indeed, liraglutide and semaglutide have demonstrated reductions in albuminuria and have been associated with a slower decline in kidney function. Thus, these medications may be double-edged arrows for the treatment of obese patients with CKD, a non-trivial proportion of the CKD population. Additionally, novel immunosuppressants like belimumab and voclosporin have come under the spotlight for the treatment of lupus nephritis [2]. The role of complement inhibitors is also becoming increasingly recognized. Drugs such as eculizumab or ravulizumab [4], which target the complement pathway, have been used successfully in treating atypical hemolytic uremic syndrome and are being explored for other forms of CKD where complement activation plays a role. Lastly, cell-based therapies, including mesenchymal stem cell (MSC) therapy, are on the horizon as a potential treatment for CKD. These therapies aim to leverage the regenerative capacity of stem cells to repair kidney damage. While still in the early stages of clinical research, they offer a novel mechanism of action that could complement existing therapies.

In conclusion, while current therapies such as SGLT2i and MRAs have significantly advanced CKD treatment, implementing them broadly and exploring new therapeutic avenues remains crucial. These emerging treatments offer hope for improved renal outcomes and highlight the importance of personalized medicine in managing CKD, as they may be more suitable for certain patient subgroups based on specific pathophysiological characteristics. The future of CKD management appears to be shifting towards personalized medicine and novel methodological approaches that may further reduce the morbidity and mortality associated with this condition.
